# Serum GP73 predicts posthepatectomy outcomes in patients with hepatocellular carcinoma

**DOI:** 10.1186/s12967-019-1889-0

**Published:** 2019-05-02

**Authors:** Meng-yun Ke, Xiao-ning Wu, Yao Zhang, Saisai Wang, Yi Lv, Jian Dong

**Affiliations:** 1 Department of Vascular Surgery, First Affiliated Hospital of Xi’an Jiaotong University, Xi’an, Shaanxi China; 2 Institute of Advanced Surgical Technology and Engineering, The First Affiliated Hospital of Xi’an Jiaotong University, 277 West Yanta Road, Xi’an, 710061 Shaanxi China; 3 National Local Joint Engineering Research Center for Precision Surgery & Regenerative Medicine, The First Affiliated Hospital of Xi’an Jiaotong University, 277 West Yanta Road, Xi’an, 710061 Shaanxi China; 4 Department of Hepatobiliary Surgery, The First Affiliated Hospital of Xi’an Jiaotong University, 277 West Yanta Road, Xi’an, 710061 Shaanxi China

**Keywords:** GP73, Hepatocellular carcinoma, Liver fibrosis, Posthepatectomy outcomes

## Abstract

**Background and aims:**

Serum GP73 is a useful biomarker in assessing hepatic fibrosis degree. The aim of this study was to evaluate the predictive value of serum GP73 level for posthepatectomy short-term outcomes in hepatocellular carcinoma (HCC) patients.

**Methods:**

A total of 280 patients undergoing liver resection for HCC between October 2015 and April 2018 were included in this study. Detailed preoperative clinicopathological data were collected and GP73 levels in serum obtained the day before hepatectomy were examined. Receiver operating characteristic (ROC) analysis was used to calculate the optimal cutoff of GP73, and independent risk factors for postoperative outcomes was assessed by logistic regression model.

**Results:**

The mean GP73 level in patients was 111.8 ± 153.3 ng/mL. Serum GP73 levels were correlated with the METAVIR fibrosis score. Overall complications occurred in 145 patients and major complications developed in 29 patients. ROC analysis demonstrated that the predictive power of serum GP73 for postoperative outcomes was greater than the Child–Pugh score, ALBI score, FIB-4 index and APRI score. The optimal value of serum GP73 to predict overall complications and major complications was 80.9 and 79.2 respectively. Serum GP73 levels were independent factors affecting the incidence of overall complications (odds ratio [OR], 3.996; 95% CI 2.152–7.421; P < 0.001) and major complications (OR, 4.970; 95% CI 1.654–14.934; P = 0.004) by multivariate analysis.

**Conclusion:**

Serum GP73 is a useful tool to stratify HCC patients and to predict short-term outcomes after hepatectomy.

**Electronic supplementary material:**

The online version of this article (10.1186/s12967-019-1889-0) contains supplementary material, which is available to authorized users.

## Background

Hepatocellular carcinoma (HCC) is one of the most common causes of death from cancer throughout the world [1]. Hepatectomy remains the most effective treatment aiming at cure. With improvements in perioperative patient care, resection technique, instrumentation, and intensive care unit management, the postoperative morbidity and mortality have decreased significantly over the decades [2]. However, at the same time, the indication for liver resection has been gradually enlarged, and posthepatectomy morbidity remains a critical issue that needs to be cautious.

In recent decades, a number of markers, including liver function tests, and imaging modalities have been developed to evaluate the risk of adverse outcomes after hepatectomy [3, 4]. However, the predictive accuracy is moderate, and a more accurate biomarker is still in strong demand by clinicians.

Liver fibrosis and cirrhosis imply impaired liver function and coagulation function as well as performance status. Prior studies have also demonstrated that liver cirrhosis affects liver regeneration after liver resection. Thus, liver cirrhosis is a noted adverse factor for morbidity and mortality after hepatectomy [5, 6]. However, a long- standing problem with surgeons is that assessing the degree of liver fibrosis without liver biopsy is difficult before surgery. In most cases, the decision of hepatectomy could only be depended on the degree of liver cirrhosis assessed by the naked eye after laparotomy.

Golgi protein 73 (GP73) is a type II transmembrane glycoprotein that resides within the cis-Golgi complex normally [7]. GP73 contains a protease cleavage site, truncated GP73 cutted by protease can be secreted into the circulation [8, 9]. The expression level of GP73 in liver tissue and serum is closely associated with liver disease, including acute hepatitis, liver cirrhosis and HCC. Recent studies have reported that GP73 is a powerful marker in predicting significant fibrosis in patients with chronic HBV infections [10, 11]. However, the function of GP73 for assessing the occurrence risk of postoperative adverse outcomes has not been elucidated. Thus, the aim of this study was to evaluate whether serum GP73 levels predict postoperative overall complications and major complications in HCC patients.

## Patients and methods

The study was approved by the Clinical Research Ethics Committee of the First Affiliated Hospital of Xi’an Jiaotong University and was exempted from the requirement to obtain informed consent. The study protocol observed the ethical standards of the Helsinki Declaration.

Between October 2015 and April 2018, 280 HCC patients with Child–Pugh grade A or B, who underwent liver resection at the Department of Hepatobiliary Surgery, the First Affiliated Hospital of Xi’an Jiaotong University, were enrolled in this study.

The clinicopathological data analyzed in the present study included gender, age, diabetes, hypertension, ASA grading, total bilirubin, albumin, ALT, AST, size of tumor, number of tumors, operation time, blood loss, and transfusion of red blood cells. The values of ALBI score, APRI score and FIB-4 index were calculated according to the original formula reported previously [12–14]. Postoperative mortality was defined as death within 90 days after liver resection. The severity of postoperative complications was assessed according to the Dindo–Clavien classification. Major complications were defined as grade 3 or above [15]. The primary outcomes of this study was overall complication and major complication, the secondary outcomes included operative blood loss, blood transfusion and hospital stay.

### Measurement of serum levels of GP73

For patients who underwent liver resection, a frozen serum sample was collected within 1 week before surgery and was stored at − 80 °C. The double-antibody sandwich enzyme-linked immunosorbent assay (ELISA) kit (Hotgen Biotech Inc., Beijing, China) was used to measure the serum GP73 values according to the manufacturer’s protocol.

### Bioinformatics analysis of Oncomine cancer gene microarray database

For analysis of GP73 mRNA expression in normal liver tissues, liver cirrhosis tissues and HCC tissues, we searched all the 8 HCC datasets from the Oncomine database, only Mas datasets which included 19 normal liver tissues, 58 liver cirrhosis tissues and 38 HCC tissues qualified for this study. This allowed us to compare GP73 transcript expression between different groups.

### Statistical analysis

All statistical analyses were done using SPSS 24.0 software (SPSS, Chicago, IL). Continuous variables are described as the mean ± standard deviation (SD), and were compared by Student’s t-test or Mann–Whitney U test, as appropriate. Categorical data are expressed as numbers and percentages, and compared with the χ^2^ test or Fisher’s exact test. MedCalc software was used to develop the receiver operating curve (ROC) and the predictive accuracy of each index was compared using the method of DeLong. The optimal cutoff of serum GP73 value predicting postoperative outcomes was obtained by the receiver operating characteristic curves. Univariate and multivariate analyses were used to identify the independent risk factors for the development of postoperative primary outcomes. Only variables significantly associated with endpoints in the univariate analysis were subjected to the multivariate logistic regression analysis. All tests of significance were two-tailed, and P < 0.05 was considered statistically significant.

## Results

### Patient characteristics

The demographic and clinical characteristics of the enrolled patients are summarized in Table 1. The mean age of these patients (238 men and 42 women) was 50.9 years. One hundred and eighty-six patients had background viral hepatitis. Among these patients, 176 were positive for the HBV surface antigen, 8 were positive for the HCV antibody, and 2 patients were positive for both the HBV surface antigen and the HCV antibody. The majority of patients (93.6%) belonged to Child–Pugh grade A and the remaining 18 had Child–Pugh class B, there was no patient with Child–Pugh class C classification.

**Table 1:** Subject characteristics

Patient characteristics	n (%)
Age, mean ± SD (year)	50.9 ± 11.5
Male, n (%)	238 (85.0%)
Diabetes, n (%)	22 (7.9%)
Hypertension, n (%)	53 (18.9%)
Background liver disease	
HB	176
HC	8
HB and HC	2
Non-B non-C	94
ASA grading, mean + SD	2.3 ± 0.6
Hemoglobin, mean ± SD (g/dL)	130.7 ± 19.7
White blood cell count, mean ± SD (10^9^/L)	5.5 ± 2.5
Platelet count, mean ± SD (10^9^/L)	135.8 ± 71.0
Total bilirubin, mean ± SD (μmol/L)	24.4 ± 45.7
ALT, mean ± SD (U/L)	54.2 ± 57.1
AST, mean ± SD (U/L)	56.8 ± 63.2
INR, mean ± SD	1.08 ± 0.1
Albumin, mean ± SD (g/L)	39.1 ± 5.5
GP73, mean ± SD (ng/mL)	111.8 ± 153.3
Child–Pugh score, mean ± SD	5.6 ± 1.0
Child–Pugh grade (A/B/C)	262/18/0
Size of largest tumor, mean ± SD (cm)	6.2 ± 3.6
Solitary tumor, n (%)	246 (87.9%)
Blood loss, mean ± SD (min)	805.8 ± 961.8
Intermittent hilar clamping, mean ± SD (min)	14.3 ± 15.2
Operation time, mean ± SD (min)	233.5 ± 200.2
Intraoperative procedures	
Minor hepatectomy	199
Major hepatectomy	81
Clavien–Dindo classification	
Grade 1	35
Grade 2	81
Grade 3a/3b	19/1
Grade 4a/4b	5/0
Grade 5	4

*AST* aspartate aminotransferase, *ALT* alanine aminotransferase, *ASA* American Society of Anesthesiology, *HBV* hepatitis B virus, *HCV* hepatitis C virus

### Perioperative data

The type of hepatectomy was anatomical liver resection, 199 patients underwent a minor hepatectomy and 81 patients underwent a major hepatectomy. The mean diameter of the largest tumors was 6.2 ± 3.6 cm, the mean operation time was 233.5 ± 200.2 min, and the mean operative blood loss was 805.8 ± 961.8 mL.

The overall complication rate was 58.0%. Among these patients, 29 patients (10.4%) had major complications, and only 4 patients (1.4%) died within 3 months after surgery. Postoperative pathologic results showed that 33 patients had no fibrosis (F0), 55 patients had mild fibrosis (F1), 72 patients had severe fibrosis (F2), 62 patients had significant fibrosis (F3) and 58 patients had cirrhosis (F4).

### Correlation of GP73 to histological fibrosis staging

**Fig. 1:**
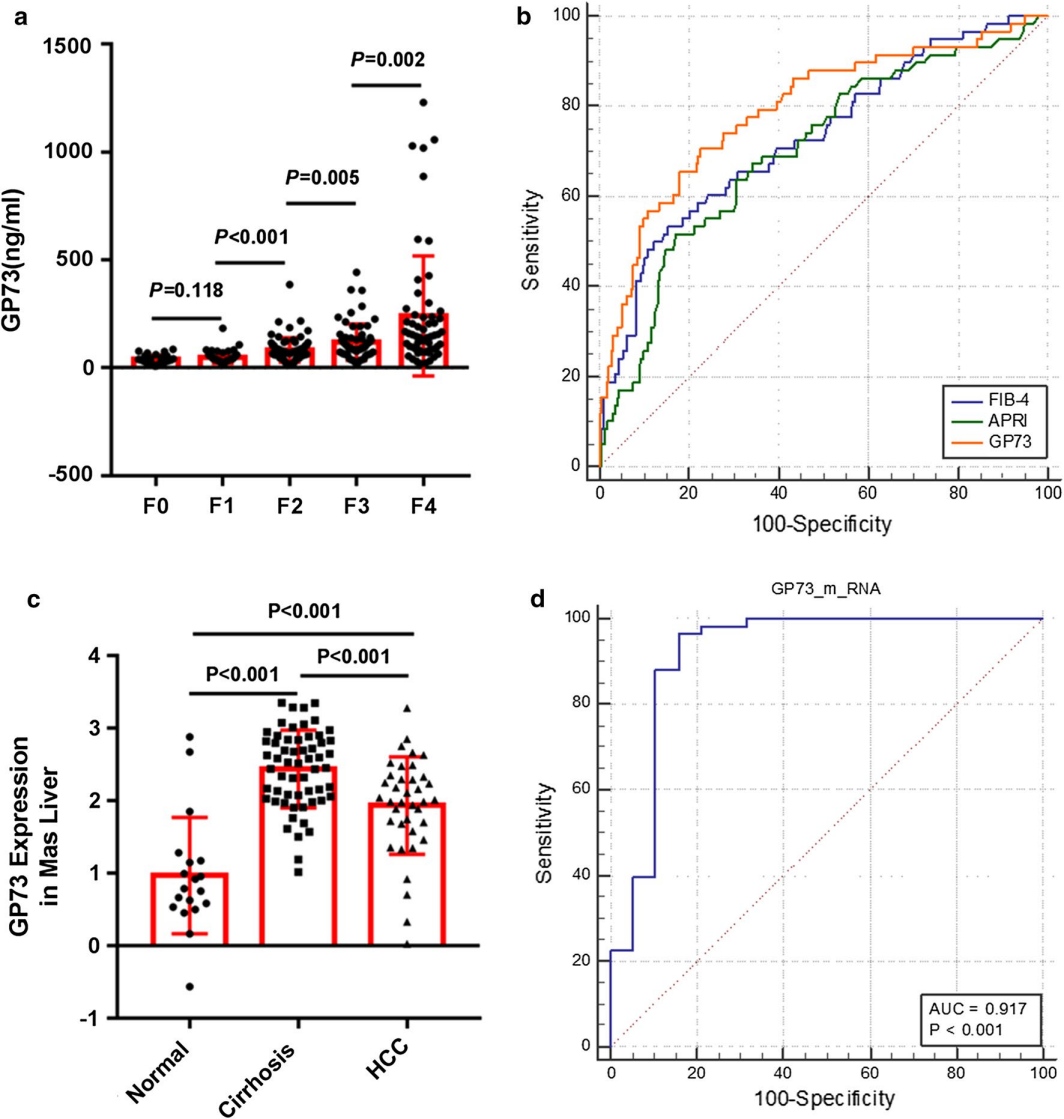
Serum GP73 values for each Metavir fibrosis stage (**a**); ROC curve for serum GP73 in relation to liver cirrhosis (**b**); GP73 mRNA levels for normal liver tissues, liver cirrhosis tissues and HCC tissues in the Mas liver dataset (**c**); ROC curve for GP73 mRNA levels in relation to liver cirrhosis in the Mas liver dataset (**d**)

A plot of serum GP73 in relation to the Metavir fibrosis stage is presented in Fig. 1a. The mean (± SD) serum GP73 values in stage F0, F1, F2, F3, and F4 patients were 39.1 ± 19.8, 47.5 ± 26.3, 83.2 ± 56.3, 119.3 ± 84.2, and 241.2 ± 278.2, respectively. There were significant differences between stage F1 and stage F2, stage F2 and stage F3, and stage F3 and stage F4. The ROC curve for serum GP73 in relation to liver cirrhosis (F4) is shown in Fig. 1b. The AUROC of serum GP73 [0.792 (95% confidence interval, 0.722–0.862; P < 0.001)] was significantly higher than those for APRI and FIB-4 (Additional file 1: Table S1).

Next, we explored the expression levels of GP73 mRNA in human liver tissues from the Oncomine database. Only the Mas liver dataset had gene expression data for normal liver tissues, liver cirrhosis tissues and HCC tissues (Fig. 1c) [16]. The dataset revealed that the levels of GP73 mRNA in HCC tissues (1.9 ± 0.7) were higher than those in normal liver tissues (1.0 ± 0.8), (P < 0.001). Moreover, the expression levels of GP73 mRNA in liver cirrhosis tissues (2.4 ± 0.5) was even higher than that in HCC tissues (P < 0.001). The ROC curve was used to assess the discriminatory power of GP73 mRNA for liver cirrhosis from normal liver. The AUROC value was 0.917 (95% confidence interval, 0.820–1.000; P < 0.001) (Fig. 1d).

### Prediction of postoperative complications

**Fig. 2:**
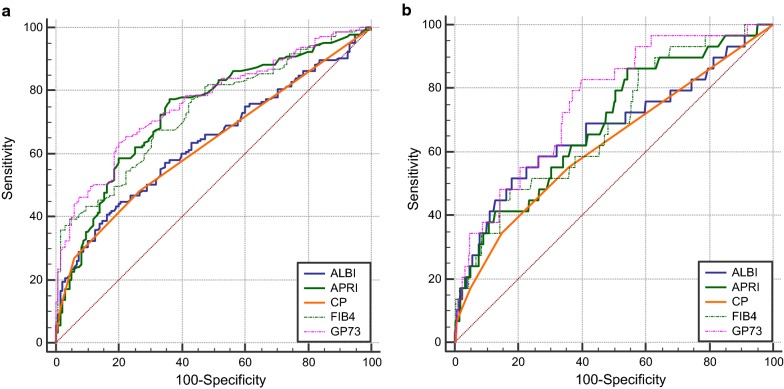
Receiver operating characteristic (ROC) curves for noninvasive markers in prediction of postoperative overall complications (**a**) and major complications (**b**)

The ROC curves for serum GP73 and the other non-invasive markers in relation to postoperative overall complications are shown in Fig. 2a. The AUROC curve was 0.763 (95% confidence interval, 0.708–0.818; P < 0.001) for serum GP73, and the calculated cutoff value was 80.9 ng/mL with a sensitivity of 63.4%, and a specificity of 80.0% in the prediction of overall complications (Table 2). The predictive accuracy of GP73 for postoperative overall complications is greater than other several markers. However, the superiority of serum GP73 over APRI and FIB-4 were not statistically significant.

**Table 2:** Receiver operating characteristic analysis of noninvasive markers in predicting postoperative outcomes

Variable(s)	Area	*P* value	*P* value* (vs GP73)	95% CI
Overall morbidity				
GP73	0.763	< 0.001		0.708–0.818
ALBI	0.637	< 0.001	0.001	0.573–0.702
FIB-4	0.734	< 0.001	0.159	0.676–0.792
APRI	0.733	< 0.001	0.271	0.674–0.792
Child–Pugh score	0.631	< 0.001	< 0.001	0.566–0.696
Severe morbidity				
GP73	0.756	< 0.001		0.668–0.845
ALBI	0.671	0.003	0.102	0.551–0.790
FIB-4	0.684	0.001	0.037	0.580–0.787
APRI	0.687	0.001	0.155	0.584–0.790
Child–Pugh score	0.622	0.031	0.027	0.506–0.739

*P value, comparisons of respective AUROC of other method with that of GP73

The AUROC value for the serum GP73 (AUC 0.756, 95% CI, 0.668–0.845; P < 0.001) in predicting postoperative severe complications was greater than that for the other markers, but the statistically significance between GP73 and ALBI and APRI was not observed (Fig. 2b). The optimal cut-off value of serum GP73 was 79.2 ng/mL with a sensitivity of 82.8% and a specificity of 60.2% for postoperative severe complications.

**Fig. 3:**
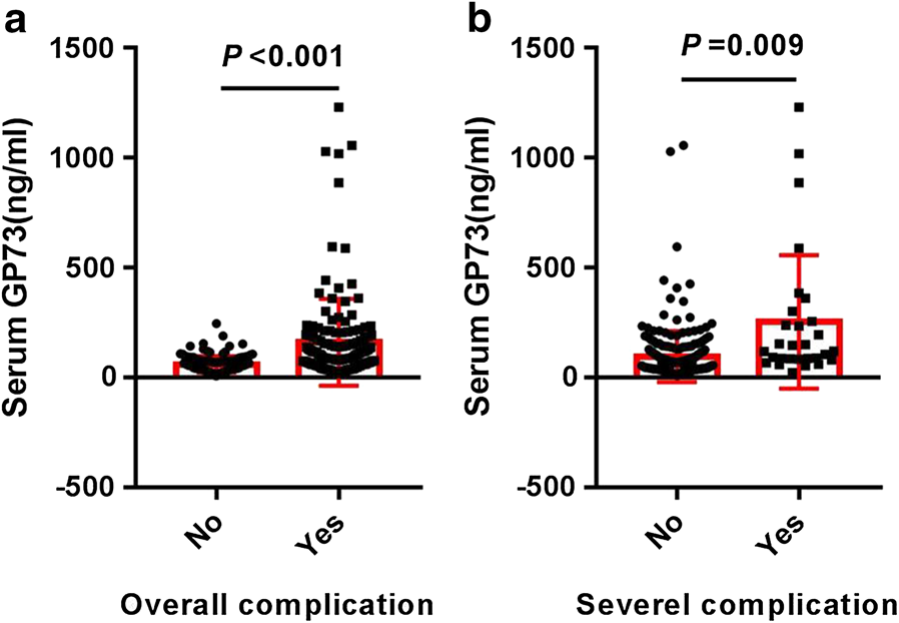
Distribution of serum GP73 values according to the occurrence of overall complications (**a**) and severe complications (**b**)

The distributions of serum GP73 values according to the occurrence of postoperative overall complication and major complication were shown in Fig. 3. The serum GP73 value of the complication-positive group was significantly higher than that of the complication-negative group.

### Risks factors of posthepatectomy outcomes

First, serum GP73 values were transformed into categorical data on the basis of the cutoff value in the logistic regression model. Univariate analysis showed that age, total bilirubin, albumin, ALT, AST, GP73, operation time, blood loss, and intraoperative red blood cell transfusion were significant predictors of overall complications after hepatectomy. In the multivariate analysis, albumin (odds ratio [OR] = 0.922; 95% confidence interval [CI] 0.871–0.975; P = 0.004), ALT (OR = 1.012; 95% CI 1.001–1.022; P = 0.026), GP73 of 80.9 ng/mL or greater (OR = 3.996; 95% CI 2.152–7.421; P < 0.001), and intraoperative red blood cell transfusion (OR = 1.206; 95% CI 1.069–1.359; P = 0.002), were independently associated with overall complications (Table 3).

**Table 3:** Univariate and multivariate analysis of postoperative overall complications

Variables	No complications	Complications	Univariate analysis	Multivariate analysis	
	135	145	*P*	OR (95% CI)	*P*
Gender (male/female)	116/19	122/23	0.675		
Age (year) (≤ 50 vs > 50)	69/66	62/83	*0.027*		
Diabetes (yes vs no)	9/126	13/132	0.475		
Hypertension (yes vs no)	24/111	29/116	0.635		
ASA grading (≤ 2 vs > 2)	101/34	94/51	0.069		
Total bilirubin (μmol/L)	15.2 ± 8.0	33.0 ± 61.9	*0.001*		
Albumin (g/L)	40.7 ± 5.1	37.7 ± 5.5	*< 0.001*	0.922 (0.871–0.975)	*0.004*
ALT (U/L)	38.0 ± 23.0	69.4 ± 73.3	*<0.001*	1.012 (1.001–1.022)	*0.026*
AST (U/L)	37.7 ± 19.7	74.4 ± 81.9	*< 0.001*		
GP73 (≤ 80.9 vs > 80.9 ng/mL)	108/27	53/92	*< 0.001*	3.996 (2.152–7.421)	*< 0.001*
Size of tumor (cm)	6.1 ± 3.4	6.3 ± 3.8	0.749		
Number of tumors	1.1 ± 0.4	1.2 ± 0.5	0.521		
Operation time (min)	200.9 ± 65.4	263.9 ± 267.9	*0.008*		
Blood loss (mL)	546.1 ± 510.5	1051.0 ± 1197.5	*< 0.001*		
Intraoperative RBC transfusion	1.9 ± 2.0	3.8 ± 4.3	*< 0.001*	1.206 (1.069–1.359)	*0.002*

*AST* aspartate aminotransferase, *ALT* alanine aminotransferase, *ASA* American Society of Anesthesiology, *RBC* red blood cell

Albumin, ALT, AST, GP73, blood loss, and intraoperative red blood cell transfusion were significantly related to postoperative major complications at univariate analysis. Three independent predictors of major complications were identified at multivariate analysis: ALT (OR = 1.006; 95% CI 1.001–1.011; P = 0.019), GP73 of 79.2 ng/mL or greater (OR = 4.970; 95% CI 1.654–14.934; P = 0.004), and blood loss (OR = 1.001; 95% CI 1.000–1.001; P < 0.001; Table 4).

**Table 4:** Univariate and multivariate analysis of postoperative severe complications

Variables	No complications	Complications	Univariate analysis	Multivariate analysis	
	251	29	*P*	OR (95% CI)	*P*
Gender	217/34	21/8	*0.045*		
Age (year) (≤ 50 vs > 50)	117/134	14/15	0.865		
Diabetes (yes vs no)	19/232	3/26	0.486		
Hypertension (yes vs no)	46/205	7/22	0.449		
ASA grading (≤ 2 vs > 2)	178/73	17/12	0.173		
Total bilirubin (μmol/L)	20.6 ± 33.5	57.6 ± 97.3	0.052		
Albumin (g/L)	39.4 ± 5.6	36.9 ± 4.1	*0.021*		
ALT (U/L)	48.8 ± 42.8	100.6 ± 116.6	*0.024*	1.006 (1.001–1.011)	*0.019*
AST (U/L)	51.1 ± 52.7	106.2 ± 110.6	*0.013*		
GP73 (≤ 79.2 vs > 79.2 ng/mL)	151/100	5/24	*< 0.001*	4.970 (1.654–14.934)	*0.004*
Size of tumor (cm)	6.1 ± 3.5	7.3 ± 4.5	0.065		
Number of tumors	1.2 ± 0.5	1.1 ± 0.4	0.785		
Operation time (min)	230.0 ± 208.7	263.8 ± 98.6	0.390		
Blood loss (mL)	705.3 ± 789.9	1669.0 ± 1662.6	*0.004*	1.001 (1.000–1.001)	*< 0.001*
Intraoperative RBC transfusion	2.5 ± 2.9	6.0 ± 6.5	*< 0.001*		

*AST* aspartate aminotransferase, *ALT* alanine aminotransferase, *ASA* American Society of Anesthesiology, *RBC* red blood cell

### Patients characteristics according to serum GP73 levels

**Fig. 4:**
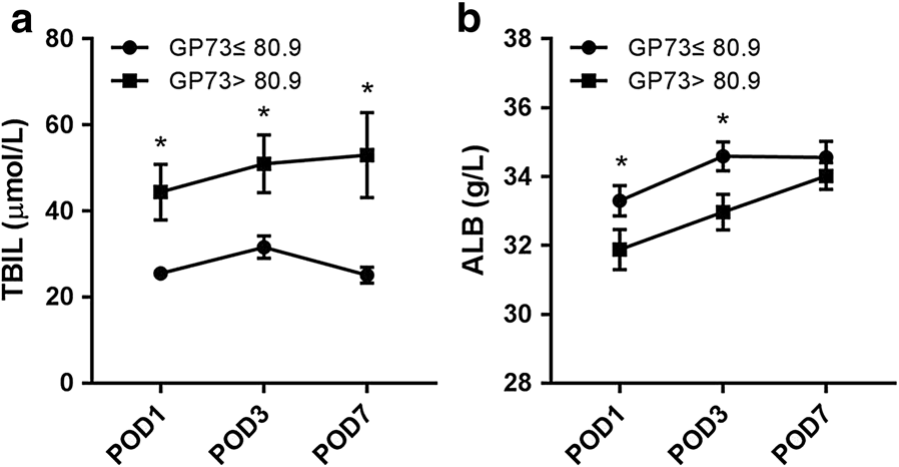
Postoperative liver injury assessed by measurements of serum total bilirubin (**a**) and albumin (**b**) for patients with GP73 > 80.9 and ≤ 80.9 ng/mL. *POD* postoperative day; *significant

Table 5 reports the characteristics of patients according to serum GP73 levels. Patients with elevated GP73 levels had a higher ASA grading, higher Child–Pugh score, and higher preoperative levels of total bilirubin, ALT, AST, albumin, white blood cell, platelet count, and INR. Besides, the intraoperative transfusion of red blood cells and fresh frozen plasma was also significantly higher in patients with GP73 > 80.9 ng/mL. After surgery, there were significant differences in complication rates, and hospital stays between patients with GP73 ≤ 80.9 and > 80.9 ng/mL. In addition, no significant differences in the tumor characteristics were observed between the two groups. Serum total bilirubin was significantly higher in patients with GP73 > 80.9 ng/mL than in patients with GP73 ≤ 80.9 on PODs (postoperative days) 1, 3, and 7 (44.4 ± 63.6 vs 25.5 ± 15.2, *P* = 0.002; 51.0 ± 67.0 vs 31.6 ± 30.1 pg/mL, *P* = 0.008; and 53.0 ± 101.6 vs 25.1 ± 19.6, *P* = 0.006; respectively). The peak of the serum total bilirubin occurred on the third postoperative day in patients with GP73 ≤ 80.9, whereas, the level of serum total bilirubin has generally increased over 7 days after the operation. The albumin level was lower in patients with GP73 > 80.9 ng/mL than in patients with GP73 ≤ 80.9 ng/mL on PODs 1 and 3 (33.3 ± 4.9 vs 31.9 ± 5.7, *P* = 0.049; and 34.6 ± 4.8 vs 33.0 ± 5.2 pg/mL, *P* = 0.015; respectively; Fig. 4).

**Table 5:** Baseline comparison between patients with GP73 > 80.9 and ≤ 80.9

Variable	GP73 ≤ 80.9 ng/mL	GP73 > 80.9 ng/mL	*P*
	(n = 161)	(n = 119)	
Age, mean ± SD (year)	51.3 ± 11.9	50.3 ± 11.0	0.972
Gender (male/female)	135/26	103/16	0.531
Diabetes (yes vs no)	13/148	9/110	0.875
Hypertension (yes vs no)	31/130	22/97	0.871
ASA grading, mean ± SD	2.2 ± 0.6	2.3 ± 0.6	*0.046*
Total bilirubin, mean ± SD (μmol/L)	16.3 ± 11.6	35.5 ± 67.3	*< 0.001*
ALT, mean ± SD (U/L)	34.8 ± 18.9	80.3 ± 77.5	*< 0.001*
AST, mean ± SD (U/L)	34.7 ± 14.8	86.8 ± 87.0	*< 0.001*
Albumin, mean ± SD (g/L)	40.1 ± 5.2	37.8 ± 5.5	*< 0.001*
Child–Pugh score, mean ± SD	5.4 ± 0.8	5.9 ± 1.1	*< 0.001*
WBC, mean ± SD (10^9^/L)	5.9 ± 2.4	4.9 ± 2.4	*0.001*
Hemoglobin, mean ± SD (g/dL)	132.3 ± 21.0	128.5 ± 17.6	0.104
Platelet count, mean ± SD (10^9^/L)	165.6 ± 73.6	95.5 ± 41.5	*< 0.001*
PT, mean ± SD	13.2 ± 1.2	13.5 ± 1.4	*0.022*
APTT, mean ± SD	37.7 ± 7.4	38.0 ± 8.2	0.758
INR, mean ± SD	1.06 ± 0.10	1.10 ± 0.11	*0.001*
Size of tumor, mean ± SD (cm)	5.9 ± 3.5	6.5 ± 3.7	0.183
Number of tumors	1.2 ± 0.5	1.2 ± 0.4	0.798
Operation time, mean ± SD (min)	217.0 ± 80.9	255.8 ± 291.6	0.109
Blood loss, mean ± SD (mL)	677.3 ± 856.3	980.1 ± 1067.9	*0.012*
Blood transfusion			
Red blood cells , mean ± SD(u)	2.3 ± 3.2	3.6 ± 3.9	*0.003*
Fresh frozen plasma, mean ± SD (mL)	224.8± 350.5	317.7± 330.3	*0.026*
Cryoprecipitation, mean ± SD (u)	0.6 ± 1.8	8.5 ± 73.6	0.245
Complication			
Overall	53/108	92/27	*< 0.001*
Severe	6/155	23/96	*< 0.001*
Total hospital stay, mean ± SD (day)	20.6 ± 6.9	24.1 ± 8.3	*< 0.001*
Postoperative hospital stay, mean ± SD (day)	13.5 ± 5.7	15.7 ± 6.1	*0.002*
Length of ICU stay, mean ± SD (day)	2.6 ± 1.5	2.8 ± 1.3	0.233

*AST* aspartate aminotransferase, *ALT* alanine aminotransferase, *ASA* American Society of Anesthesiology, *FIB-4* fibrosis score 4, *WBC* white blood cell

## Discussion

Posthepatectomy complications remains an issue that deserves concern for surgeons in clinic, and accurate evaluation of preoperative risk factors is essential to prevent postoperative morbidity and mortality.

Liver fibrosis and cirrhosis, which often accompany HCC are well-known risk factors for liver resection [17]. From the perspective of molecular biology, expression levels of many damage-associated molecular patterns are up-regulated in the progression of liver fibrosis, and these damage-associated molecular patterns make liver more sensitive to ischemia/reperfusion injury [18]. On the other hand, lower levels of hepatocyte growth factor and other transcription factors in cirrhotic livers weaken liver regeneration after liver resection [19, 20]. Hence, precise assessment of the degree of liver fibrosis is an important method to predict the operative risk.

Several non-invasive tools have been developed in the assessment of liver fibrosis [21, 22]. Among them, liver stiffness has the most efficacy. A previous study reported that the corresponding AUROC of liver stiffness for the diagnosis of cirrhosis was 0.96 in patients with HCV, 0.90 in patients with HBV, and 0.96 in patients with either alcoholic or nonalcoholic steatohepatitis [23]. The only fly in the ointment is that liver stiffness is costly and requires technical or expert knowledge. Recently, several studies have confirmed the efficacy of GP73 in assessing liver fibrosis. Cao et al. [24] demonstrated that serum GP73 is a powerful serum marker, and its diagnostic accuracy was comparable to liver stiffness and significantly higher than APRI score and FIB-4 index in antiviral-naïve chronic HBV infection. Similar to previous results, the serum GP73 level in the current study demonstrated a significant correlation with liver fibrosis. GP73 showed a high accuracy in predicting liver cirrhosis.

Based on this result, we evaluated the usefulness of serum GP73 in predicting postoperative outcomes. In the present study, multivariable analysis revealed that the serum GP73 level was an independent predictor of postoperative overall complications and major complications. Furthermore, we found that 80.9 ng/mL should be the critical value of the serum GP73 for postoperative risk-stratification. On the basis of this critical value, patients with elevated GP73 levels displayed worse liver function, coagulation function and basic status, and consequent higher blood loss, increased blood transfusion and longer hospital stays come with these patients. Furthermore, patients with elevated GP73 levels showed a slower recovery of liver function after surgery. In the end, the overall and major complication rates were significantly higher in patients with GP73 > 80.9 ng/mL. The cutoff value at 80.9 ng/mL was helpful in risk stratification for patients with hepatocellular carcinoma.

The Child–Pugh score is a traditional liver function index which has been widely used in clinic. The albumin–bilirubin (ALBI) score which is another new liver serve maker reported by Johnson and colleagues, it has been validated to be a useful tool to assess the posthepatectomy short-term and long-term prognosis for HCC patients [25–27]. In addition, other noninvasive liver fibrosis tools including FIB-4 index and APRI score also have the power to evaluate liver function reserve. Assessment of liver function is particularly important for HCC patients who are prepared for surgery, because it is perceived that liver cirrhosis is a major cause of death. Our results demonstrated that the serum GP73 level had a greater discriminatory power than the other noninvasive models in predicting postoperative overall complications and major complications. Thus, serum GP73 is a excellent tool that can reflect the liver function of HCC patients very well.

Recently, researches reported that serum GP73 is a potential diagnostic marker for HCC [28, 29]. However, some other studies showed that the elevated levels of serum GP73 in HCC patients were mainly due to the background of liver cirrhosis [30]. In the present study, the Oncomine dataset showed that the GP73 expression levels in the liver cirrhosis tissues were the highest among the normal liver tissues, liver cirrhosis tissues and HCC tissues. In addition, the tumor characters including tumor size and tumor number showed no significant differences between patients with GP73 ≤ 80.9 and > 80.9 ng/mL. A study by Liu et al. [31] also demonstrated that serum GP73 increased in HCC patients with cirrhosis but not in those without cirrhosis, and all these results suggested that the background of cirrhosis but not HCC was related to the upregulation of serum GP73.

In the current study, GP73 did not improve prediction of postoperative outcomes significantly compared with traditional markers. However, as we often encounter a difficult situation that various traditional liver function tests show inconsistent results in judging actual liver function and we suppose that measuring GP73 in addition to conventional liver function markers is helpful for our judgment especially in such situations.

The present study has several limitations. First, the absence of liver stiffness measurements makes it impossible to compare the diagnostic accuracy in predicting postoperative outcomes between serum GP73 and liver stiffness. Second, the majority of patients had a background of hepatitis B virus, which is different from Western countries where the etiology is predominantly hepatitis C virus, other etiological cases are required to validate the application ability of GP73. Third, HCC patients with Child–Pugh grade C were excluded for liver resection at the local centre, only 4 (1.42%) patients died within 3 months after surgery, and the sample size and the number of events were relatively small. Futhermore, there was a lack of validation of the results with an independent population. Thus, a multicenter prospective study is needed to assess the efficacy of GP73 in predicting postoperative outcomes. However, even with these limitations, to our knowledge, the role of preoperative serum GP73 to predict the short-term outcomes after hepatectomy in HCC patients has never been previously explored, and to date, it is the first time for this study to report a clear correlation between preoperative serum GP73 and postoperative complication.

## Conclusion

In conclusion, our study demonstrated that GP73 is a valuable serum marker that can be used clinically to stratify patients with a high risk of postoperative complications by reflecting the status of liver fibrosis. Measurements of GP73 will also help to select the right HCC patients to undergo surgical treatment. Therefore, we recommend measuring serum GP73 levels as a routine examination for HCC patients undergoing hepatectomy. It will be interesting to combine GP73 with other markerss to seek further improvement of accuracy in future investigations.

## Additional file

**Additional file 1: Table S1.** Receiver Operating Characteristic Analysis of Noninvasive Markers in Diagnosing Liver Cirrhosis.
