# Supplementary material for: Serum GP73 predicts posthepatectomy outcomes in patients with hepatocellular carcinoma
Source: J Transl Med. 2019 May 2;17:140. doi: 10.1186/s12967-019-1889-0 (PMC6498666; doi:10.1186/s12967-019-1889-0)
Supplement: Supplementary file 1 — Additional file 1: Table S1. Receiver Operating Characteristic Analysis of Noninvasive Markers in Diagnosing Liver Cirrhosis. [file 12967_2019_1889_MOESM1_ESM.docx]

**Table S1.** Receiver Operating Characteristic Analysis of Noninvasive Markers in Diagnosing Liver Cirrhosis

| Variable(s) | Area | *P* value | *P* value^*^  ( vs GP73 ) | 95% CI |
| --- | --- | --- | --- | --- |
| GP73 | 0.792 | <0.001 |  | 0.722-0.862 |
| FIB-4 | 0.730 | <0.001 | 0.005 | 0.654-0.805 |
| APRI | 0.701 | <0.001 | 0.006 | 0.624-0.778 |
